# Supplementary material for: A qualitative description of HIV testing and healthcare experiences among trans women in Ghanaian urban slums BSGH-012
Source: PLOS Glob Public Health. 2024 Dec 2;4(12):e0003980. doi: 10.1371/journal.pgph.0003980 (PMC11611076; doi:10.1371/journal.pgph.0003980)
Supplement: S1 Data — This dataset includes anonymized data on positive and negative experiences, healthcare interactions, and demographic details, collected during face-to-face interviews. (DOCX) [file pgph.0003980.s001.docx]

| **Variables** | **Details** | **N** | **%** | **Mean** | **Min** | **Max** | **Standard Deviation** |
| --- | --- | --- | --- | --- | --- | --- | --- |
| **Age** |  | 20 | 100% |  |  |  |  |
|  | In years | 20 | 100% | 20 | 18 | 31 | 3.25 |
| **Education** |  | 20 | 100% |  |  |  |  |
|  | Tertiary education | 10 | 50% |  |  |  |  |
|  | Secondary education | 5 | 25% |  |  |  |  |
|  | primary education | 4 | 20% |  |  |  |  |
|  | No formal education | 1 | 5% |  |  |  |  |
| **Religiosity** |  | 20 | 100% |  |  |  |  |
|  | Christian | 15 | 75% |  |  |  |  |
|  | Atheist | 4 | 20% |  |  |  |  |
|  | Muslim | 1 | 5% |  |  |  |  |
| **Marital Status** |  | 20 | 100% |  |  |  |  |
|  | Single | 20 | 100% |  |  |  |  |
|  | Married |  |  |  |  |  |  |
|  | Divorce |  |  |  |  |  |  |
| **Employment Status** |  | 20 | 100% |  |  |  |  |
|  | Employed | 20 | 100% |  |  |  |  |
|  | Unemployed | 0 | 0% |  |  |  |  |
| **Average Monthly Income** |  | 20 | 100% |  |  |  |  |
|  |  |  | 100% | GH₵830.15 ($70) | GH₵200 ($14) | GH₵2003 ($132) | GH₵450.75 ($30) |
| **Sexual Behavior** |  | 20 | 100% |  |  |  |  |
|  | Exclusively having male sexual partners | 19 | 95% |  |  |  |  |
|  | Reported involvement with both male and female sexual partners | 1 | 5% |  |  |  |  |
| **Ethnicity** |  | 20 | 100% |  |  |  |  |
|  | Ewe | 2 | 10% |  |  |  |  |
|  | Akan | 6 | 30% |  |  |  |  |
|  | Ga | 8 | 40% |  |  |  |  |
|  | Ashanti | 4 | 20% |  |  |  |  |
| **Type Of Housing** |  | 20 | 100% |  |  |  |  |
|  | Renting | 11 | 55% |  |  |  |  |
|  | Own Housing | 7 | 35% |  |  |  |  |
|  | Squatting | 2 | 10% |  |  |  |  |
