# Supplementary material for: A qualitative description of HIV testing and healthcare experiences among trans women in Ghanaian urban slums BSGH-012
Source: PLOS Glob Public Health. 2024 Dec 2;4(12):e0003980. doi: 10.1371/journal.pgph.0003980 (PMC11611076; doi:10.1371/journal.pgph.0003980)
Supplement: Attachment — Submitted filename: Response to Reviewers_HDD (1).docx [file pgph.0003980.s002.docx]

**Response to Reviewers: A qualitative description of HIV testing and healthcare experiences among trans women in Ghanaian urban slums BSGH-011**

We are grateful to the editor and reviewers for their feedback and helpful recommendations. We are glad to have the chance to resubmit an improved version of our paper for your further evaluation. In the following, we address the reviewers’ remarks and indicate the relevant modifications we applied to the paper. The page numbers we refer to are from the revised manuscript with track changes.

**Reviewer #1:**

**Minor Issues:**

1. **Issue:** The submitted version still contained some edits marked by the word change control. As this is the first submission, this should not have happened, as it could confuse the reviewer.

**Response:** This has been duly rectified.

2. **Issue:** To distinguish the titles of each section from the subtitles, I suggest that they be presented in capital letters in bold (INTRODUCTION, METHODS, RESULTS, DISCUSSION, CONCLUSION) and the subtitles remain in lower case letters in bold. Alternatively, leave the subtitles in italics without bold. I recommend checking the magazine's instructions for specific guidance. Otherwise, refer to articles already published in this magazine to see how the titles and subtitles of each section are presented.

**Response:** All titles have been modified to appear in capital letters.

3. **Issue:** I suggest including the term ‘transgender women’ in the first paragraph of the introduction, along with the acronym (TGW). This acronym only appears on page 2 (third paragraph). If you prefer not to use an acronym, I suggest standardizing the term ‘trans women’ throughout the manuscript.

**Response:** The term ‘transgender women’ along with the acronym TGW has been included in the first paragraph. TGW has been used consistently throughout the text.

4. **Issue:** The same suggestion applies to the acronym LGBT – spell it out the first time it appears and standardize the acronym throughout the text. It currently appears in two forms: LBGT | LGBTQ+.

**Response:** The full meaning of LGBT has been spelled out the first time it appears, and the acronym has been standardized throughout the text.

5. **Issue:** The term "Men who have sex with men" appears in full in the Discussion when an acronym for this term (MSM) has already been included in previous sections.

**Response:** This has been corrected.

**INTRODUCTION**

It is well described and linked, however, to make the scenario related to the study population in Ghana more evident to the reader, it would be interesting to inform:

1. Is there demographic data on the number of trans people living in the country?

**Response**: Currently, there is no official or comprehensive demographic data on the number of transgender people living in Ghana

2. Are there more up-to-date epidemiological data to insert to replace reference number 2, which is from 2019? Is there a notification system to register new HIV cases, according to gender identity? Does the Ghanaian Ministry of Health prepare and make available epidemiological bulletins on HIV, which include the trans population?

**Response**: Finding up-to-date epidemiological data, particularly concerning the transgender population in Ghana, is challenging due to limited data collection and reporting specific to gender identity. Ghana does not currently have a notification system that registers new HIV cases according to gender identity. HIV data collection is usually categorized by sex (male or female) rather than gender identity. The Ghanaian Ministry of Health, through the Ghana Health Service and the National AIDS/STI Control Programme (NACP), does prepare and make available epidemiological bulletins on HIV. However, these bulletins generally do not include data disaggregated by gender identity. They mainly focus on overall HIV prevalence, incidence, and key populations as defined by behavior rather than gender identity.

3. How are health services distributed in Ghana? Are they public or proven? In what type of health service is it possible to carry out an HIV test in the country? Is there a specific network of services to provide Comprehensive Health for Trans People? Are these services characterized by being trans-friendly based on any specific training of health professionals? ‘Not trans-friendly’ health services are mentioned – what criteria are used for this definition?

**Response**: Health services in Ghana are delivered through a mix of public and private institutions. Public services, managed by the Ghana Health Service (GHS) and the Ministry of Health (MOH), are available at various levels, including teaching hospitals, regional and district hospitals, polyclinics, and community health centers. Private facilities also provide a wide range of services, including HIV testing.

HIV testing is accessible in public health facilities and many private clinics, with the latter often offering more privacy at a cost. However, there is no formal, nationwide network specifically for transgender individuals. Transgender people typically rely on general health services, which may not be adequately equipped or trained to meet their specific needs.

Most health services in Ghana are not welcoming to key populations, including trans persons with only limited initiatives by NGOs and international organizations to train healthcare providers in key population health. These efforts are not yet integrated into standard medical or nursing education.

Regarding the term "not trans-friendly," this has been revised to "not oriented towards the needs of key populations" to better reflect the criteria used for this definition.

4. As for the subtitle 'Theoretical framework', I think it could be removed, however when mentioning the gender-affirmative model, it would be interesting to provide more information about it and how its principles connect with the objective of expanding the search for health services for testing of HIV.

**Response**: The theoretical framework has been revised to provide more detailed information about the gender-affirmative model and its principles. The revised section now explicitly connects these principles with the objective of expanding access to HIV testing services for transgender women.

**METHODS**

5. Research design – I suggest inserting the bibliographic reference on qualitative research.

**Response**: A bibliographic reference on qualitative research has been inserted

6. Sampling and Recruitment procedure – the use of the snowball sampling approach was mentioned. It is necessary to better explain how this technique was applied in practice. Was a map of existing slums in Accra used? Were participants from different favelas or just one? Was there already a previous connection between them and the researchers? Was a prior questionnaire administered to confirm the eligibility criteria? Inform whether the participants received financial compensation to cover transportation and food costs and what was the amount?

**Response**: We have revised the manuscript to provide a more detailed explanation of how the snowball sampling technique was applied in practice on page 4

7. Data collection procedure – insert bibliographic references when mentioning the saturation issue to define the number of participants.

**Response**: A bibliographic reference on saturation has been inserted on page 5

8. Nature of questions - Inform the content of the Guide prepared and whether there was a script to support the interviewers and which questions made up this script, in addition to the examples cited.

**Response**: The nature of the questions has been updated as requested. The content of the interview guide has been detailed, and it has been clarified that a semi-structured script was included to support interviewers on page 5

9. Analytical strategy – explain more about the ‘meticulous approach’ and insert bibliographic references.

**Response**: This phrase has been modified to “systematic approach,” which is what we intended.

**RESULTS**

10. The findings are very interesting and their description would benefit if an additional synthetic table was presented with the identified categories and subcategories.

**Response**: A synthetic table presenting the identified categories and subcategories has been created.

11. The first paragraph of the subitems Positive and Negative - Experiences with HIV Testing are unnecessary, as they are already included in the Description of Categories and Subcategories.

**Response**: The paragraphs of the subitems have been deleted

12. In relation to the three people who already had HIV, it would be important to know how long ago they became aware of their status, as the quality of care in the health services used may have changed during this period. In this sense, would it be appropriate to address issues relating to access barriers and quality of care separately for these participants? I suggest saying something about it in the Discussion section.

**Response**: Thank you for this valuable suggestion. While understanding how the duration of HIV awareness affects care quality is an important area of research, it falls outside the scope of this study which is focused on providing a qualitative description of HIV testing experiences among transgender women in Ghanaian slums. It specifically aims to capture their immediate experiences during the testing process, rather than delving into the longitudinal aspects of HIV care and the evolution of service quality over time. Addressing issues related to access barriers and quality of care for those who have been living with HIV for some time would indeed be valuable, but such an analysis would require a different study design and focus. For a comprehensive exploration of these aspects, future research could consider longitudinal studies or analyses that specifically target the evolution of care quality and access over time.

13. When presenting excerpts of the speeches, it is not necessary to identify each of them as trans women, as this does not distinguish them. Only one was identified as a ‘Muslim’, wouldn’t that make her identification easier? There are also two Christian participants aged 26, they are also indistinguishable. It would be necessary to create another form of identification.

**Response**: Addressed. We have used participant identifiers, along with age and religion, to distinguish the excerpts.

**DISCUSSION**

14. The main findings were discussed in light of the literature and compared with the results of other research. Many points of similarity were found. There are some results that emerged in contrast to the literature. If so, it would be important to highlight it as an additional contribution of this study.

**Response**: Addressed. The positive experiences reported by TGW in this study are in contrast to what has been found in other African contexts, and this has been highlighted in the discussion section as an additional contribution of our research.

15. However, I missed discussing these data considering the reality of health services in terms of management. Are there government initiatives that are already underway and that involve health services? Are there government publications that explain what has been planned or is already underway to reduce the problems identified?

**Response**: No, there are no government initiatives or publications specifically addressing the issues identified in this study. This is largely because the group is often marginalized due to pervasive stigma and discrimination, and as a result, the government does not recognize them or target initiatives to address their specific needs.

16. Every time the gender affirmation model is cited, its respective reference must be inserted. It would also be necessary to insert bibliographic references in the fourth paragraph (open communication).

**Response**: This has been addressed. The gender affirmation model has been cited with its respective reference throughout the text, and bibliographic references have been inserted in the fourth paragraph, particularly in the section discussing open communication

17. Strengths and Limitations – should come before the Conclusion section

**Response**: This has been addressed

**Reviewer #2:**

This is a well-written paper about barriers and facilitators to HIV testing among transgender women who reside in slums in the greater Accra area. The paper is interesting and informative, and I appreciate the opportunity to review it. I have only a few recommendations for improvement:

1. The Introduction is very comprehensive and well-written. The authors may wish to include somewhere in the introduction or in the results a Figure depicting the gender affirmative model and how it was used to guide the theoretical framework for this paper. It could be a good addition for readers to have a visual of how each facilitator and barrier identified fit into this model, if you agree this makes sense.

**Response**: We really appreciate the reviewer’s suggestion for including a figure that depicts the Gender Affirmation Model. And will consider incorporating such suggestion in our future papers.

2. On page 9, it seems there is a quote missing or some editing that needs to happen as the sentence ends with “states.” For both the sections on Inclusive, Informative and Supportive Counseling and the section on Fear and Anxiety, it would be useful to break up the descriptive paragraphs and put each sentence before the corresponding quote. I think this would make the sections read more smoothly.

**Response**: This has been addressed and the descriptive paragraphs in the sections on "Inclusive, Informative and Supportive Counseling" and "Fear and Anxiety" have been reorganized. Each sentence now precedes the corresponding quote, improving the flow and readability of these sections

3. The main addition that could greatly improve the paper is if the authors included more information about the testing sites (not sure if this information was gathered, but if so, it would be very useful). For example, at testing sites where participants had positive experiences, was there anything they had in common contrasted with those where participants had negative experiences? One participant makes reference to the different care she received at an Outpatient unit. I am wondering if people go to a local community testing center versus a larger hospital, etc if the care they receive is different. This could also inform policies and guidelines for testing based on type of facility.

**Response**: Respondents indeed reported experiencing negative experiences at non-key population (KP) friendly sites, while some participants highlighted positive experiences in more inclusive settings. This difference in experiences has been discussed in the paper, particularly in the context of how KP-friendly facilities, such as the West Africa AIDS Foundation (WAAF) and the International Health Clinic Center (IHCC), offer more supportive and affirming care compared to general healthcare facilities

4. Finally, I wonder about the availability of self-testing in Ghana. Is it available at all? If so, could that be another option for those who describe that the atmosphere of testing sites and witnessing others get their results increases fear and anxiety?

**Response**: HIV self-testing is relatively new in Ghana but is gradually gaining awareness. The National AIDS Control Program (NACP) has even released implementation guidelines to support its rollout. This could indeed be a viable option for participants who experience fear and anxiety in traditional testing environments. We have highlighted this potential in the discussion section of the paper.
